# Supplementary material for: Reliability and Validity of the Emotional Eater Questionnaire in Romanian Adults
Source: Nutrients. 2022 Dec 21;15(1):26. doi: 10.3390/nu15010026 (PMC9823580; doi:10.3390/nu15010026)
Supplement: Supplementary file 1 [file nutrients-15-00026-s001.zip › nutrients-2098045-supplementary.pdf]

Table S1: The Emotional Eater Questionnaire (English/Romanian version)
Putnoky et al
Emotional Eater Questionnaire – Romanian validation

No	Questions	Întrebări
1	Do the weight scales have a great power over you? Can they change your mood?	Cântarul are o mare putere asupra ta? Îți poate schimba starea de spirit?
2	Do you crave specific foods?	Poftești anumite alimente?
3	Is it difficult for you to stop eating sweet things, especially chocolate?	Îți este greu să nu mai consumi dulciuri, în special ciocolată?
4	Do you have problems controlling the amount of certain types of food you eat?	Ai probleme în a controla cantitatea anumitor tipuri de alimente pe care le consumi?
5	Do you eat when you are stressed, angry or bored?	Mănânci când ești stresat, furios sau plictisit?
6	Do you eat more of your favourite food and with less control when you are alone?	Mănânci mai mult din mâncarea preferată și cu mai puțin control atunci când ești singur?
7	Do you feel guilty when eat "forbidden" foods, like sweets or snacks?	Te simțiți vinovat când mănânci alimente „interzise", cum ar fi dulciurile sau gustările?
8	Do you feel less control over your diet when you are tired after work at night?	Ai mai puțin control asupra ceea ce mănânci când ești obosit seara, după muncă?
9	When you overeat while on a diet, do you give up and start eating without control, particularly food that you think is fattening?	Când ești la dietă, se întâmplă să renunți și să începi să mănânci fără control, în special alimente despre care crezi că îngrașă?
10	How often do you feel that food controls you, rather than you controlling food?	Cât de des simți că mâncarea te controlează, în loc să controlezi tu mâncarea?
